# Enhancing Sodium-Ion Energy Storage of Commercial Activated Carbon by Constructing Closed Pores via Ball Milling

**DOI:** 10.3390/nano14010065

**Published:** 2023-12-26

**Authors:** Xiaojie Wang, Qian Fang, Tiejun Zheng, Yanyan Xu, Rui Dai, Zhijun Qiao, Dianbo Ruan, Yuzuo Wang

**Affiliations:** Institute of Advanced Energy Storage Technology and Equipment Faculty, Ningbo University, Ningbo 315211, Chinaqiaozhi8702@163.com (Z.Q.)

**Keywords:** activated carbon, mechanical ball-milling, closed pores, sodium-ion batteries

## Abstract

Mechanical ball milling is a prevalent technology for material preparation and also serves as a post-treatment method to modify electrode materials, thus enhancing electrochemical performances. This study explores the microstructure modification of commercial activated carbon through mechanical ball milling, proving its efficacy in increasing sodium-ion energy storage. The evolution of activated carbon’s physical and chemical properties during ball milling was systematically examined. It was observed that the quantity of closed pores and the graphitization degree in activated carbon increased with extended ball milling duration. The sodium storage mechanism in activated carbon transitions to an insertion-pore filling process, significantly elevating platform capacity. Additionally, ball-milled activated carbon demonstrates remarkable long-term cycling stability (92% capacity retention over 200 cycles at 200 mA g^−1^) and rate performance. This research offers a novel approach to developing advanced anode materials for sodium-ion batteries.

## 1. Introduction

Sodium-ion batteries (SIBs) are emerging as a viable alternative for large-scale energy storage due to sodium’s abundance, affordability, and accessibility [1,2,3,4,5,6]. However, advancing high-performance electrode materials remains a pivotal challenge for SIBs [7,8,9]. The commercial success of SIBs largely hinges on creating high-performance, cost-effective electrode materials [10,11,12,13,14,15,16]. Anode materials for SIBs are differentiated according to the electrochemical mechanism used to store sodium ions and include insertion (carbon-based and other materials), conversion-reaction (transition-metal compounds), alloying (Bi, Sb, Sn, Ge, Pb, P), conversion-alloying dual-reaction, and organic materials [17,18]. Inserted materials usually show a relatively low theoretical specific capacity [18]. Converted, alloyed, and converted-alloyed materials with high specific capacity are promising candidates for SIBs. However, they face the challenges of extended cycle life and improved rate performance due to large structural variations and weak kinetic behavior [19,20,21]. Organic anode materials are low-cost and non-polluting, but they are easily dissolved in organic electrolytes and have poor cycle stability [22]. Recent focus has centered on carbon-based materials, particularly hard carbon, due to its short-range microstructure and larger interlayer spacing, facilitating greater sodium insertion [23,24,25,26].

As a disordered non-graphitic carbon, hard carbon demonstrates notable characteristics, including high reversible capacity, low operational voltage, prolonged cycle life, and cost-effectiveness, making it a promising candidate for SIB anodes [27,28]. Hard carbon typically consists of an internal microstructure characterized by randomly oriented turbostratic layers and domains of pseudo-graphite [29,30]. As the negative electrode in SIBs, the charge-discharge curves of hard carbon usually display a sloping region (>0.1 V) and a plateau region (<0.1 V). Current research efforts focus on increasing the closed-pore content of hard carbon to enhance the capacity of the platform segment. This is due to its higher specific capacity and lower working potential, which contribute to improved energy density in sodium-ion batteries [31,32,33].

The engineering of closed pores is generally achieved through precursor selection, carbonization process control, or chemical vapor deposition. Meng et al. constructed closed porous structures in carbon materials to enhance sodium storage capacity, utilizing ethanol as a pore-forming agent. This agent generated steam during the solvent-thermal process, leading to cavity formation between cross-linked matrices, which transformed into closed pores during carbonization [34]. Wang et al. applied activated anthracite, followed by high-temperature carbonization. This procedure facilitated the recombination of pre-existing open pores and disordered carbon structures, yielding a multitude of closed nanoscale pores within the carbon framework [35]. Chen et al. pioneered a space-enclosed chemical vapor deposition (SC–CVD) technique that integrates graphite-like carbon domains into the micropores of commercially available activated carbon. This process results in the creation of a hard carbon material with a tunable microstructure [36]. However, these methods often lack precise control over closed pore formation, making a simple and controllable approach essential for preparing high-capacity carbon anodes in sodium-ion batteries.

Mechanical ball-milling, known for its technological maturity and low cost, is frequently used for large-scale material preparation, crushing, and solid-state mixing [37,38,39]. The rapid development of mechanochemistry has expanded ball milling applications as a straightforward post-treatment method for modifying electrode materials, thus enhancing electrochemical performances [40]. Driscoll et al. noted that ball milling Li_2_MoO_4_ under high pressure results in a polymorphic phase transition to spinel, attributed to localized heating and pressure from milling ball impacts [41]. This method can induce high-pressure polycrystalline structures or transformation phases exclusive to ball milling. Ball milling also alters the microstructure of carbon materials [42,43]. Yuan et al. utilized ball milling to repair defects and introduced oxygen-containing functional groups into activated carbon. The energy storage mechanism transitioned from a double-layer capacitance to a pseudocapacitance, demonstrating significantly improved volumetric capacitance and outstanding rate performance [44].

In this study, mechanical ball milling was explored for closed-pore engineering in activated carbon, aiming to modify its pore structure for use as a high-capacity anode in sodium-ion batteries. Samples underwent mechanical ball milling and subsequent heat treatment. The BET characterization showed significant changes in the activated carbon: micropores and mesopores nearly vanished after 60 h of ball milling, and the specific surface area and pore volume were reduced to 19.61 m^2^ g^−1^ and 0.042 cm^3^ g^−1^, respectively. The true density and closed-pore capacity also varied with increased ball milling duration. In addition, the graphitization degree in activated carbon increases with extended ball milling duration. The platform capacity at voltages below 0.1 V rose from 16 mAh g^−1^ to 126 mAh g^−1^, exhibiting remarkable rate performance (112 mAh g^−1^ at 200 mA/g) and cycling stability (92% capacity retention after 200 cycles). These findings suggest that mechanical ball milling is an effective method for preparing closed pores, and its simplicity is likely to encourage broader applications and adoption.

## 2. Experimental Section

Materials Synthesis: The sample underwent mechanical milling using a planetary ball mill (TJX-4100, Dongfang Tianjing, Tianjing, China). In this process, 1 g of activated carbon (AC, YP-50F, Kuraray Co., Ltd., Tokyo, Japan) and 200 g of agate balls were placed in a 500 mL agate grinding jar. Milling was conducted at a rotational speed of 500 rpm for durations ranging from 0 to 72 h. The resulting samples were denoted as BAC-X, where X represents the milling time. A secondary heat treatment (1000~1400 °C) was then performed for 4 h in a nitrogen atmosphere to remove oxygen functional groups introduced during the ball milling process. These samples were labeled as BAC-X-Y, where Y indicates the temperature of the secondary heat treatment.

Materials Characterizations: Sample morphology was examined using a scanning electron microscope (SEM, Hitachi US-70, Tokyo, Japan). The crystal characteristics of the prepared materials were assessed through X-ray diffraction (XRD, D8 Advance, Karlsruhe, Germany). Raman spectra were obtained with a 785 nm laser on a Raman spectrometer (RENISHAW PU inVia, Wotton-under-Edge, UK). Nitrogen adsorption-desorption analysis (Micromeritics APSP 2460, Norcross, GA, USA) at –196 °C was used to analyze porosity and other detailed parameters. The specific surface area was calculated using the Brunauer–Emmett–Teller (BET) equation, and the pore size distribution was determined using the Non-Local Density Functional Theory (NLDFT) method. The true density of the materials was measured using helium gas on an AccuPyc II 1340 instrument. Surface chemical properties were characterized via X-ray photoelectron spectroscopy (XPS) on a Thermo ESCALAB 250XI instrument. For non-in situ XRD measurements, button cells were disassembled in a glovebox filled with argon gas; electrodes were washed in dimethyl carbonate (DMC), dried, and tested.

Electrochemical Measurements: CR2025 coin cells were utilized for electrochemical measurements. The slurry, composed of 80 wt% active material, 10 wt% carbon black, and 10 wt% polyvinylidene fluoride (PVDF), was coated onto a copper foil using N-methyl-2-pyrrolidone (NMP) as the solvent. The electrode was then vacuum-dried at 110 °C for 8 h to prepare the working electrode. The average loading mass of active materials was ≈1.2 mg cm^−2^. A glass fiber (Whatman, GF/D) served as the separator, and sodium metal foil was used as the counter electrode. The electrolyte comprised 1 M NaPF_6_ in a 1:1 volume mixture of ethylene carbonate (EC) and dimethyl carbonate (DMC). Assembly of the CR2025 coin cells was completed in a glovebox filled with argon gas. Electrochemical performance tests were conducted at room temperature. Cyclic voltammetry (CV) and electrochemical impedance spectroscopy (EIS) were assessed using a Bio-logic VMP3 electrochemical workstation (Seyssinet-Pariset, France). Constant current charge-discharge, rate performance, cycling performance, and galvanostatic intermittent titration technique (GITT) tests were performed on a LAND CT2001A testing instrument (Wuhan, China). For GITT, a 30 min discharge/charge process at a 0.1C (1C = 300 mA/g) current rate was followed by 180 min of rest to achieve quasi-equilibrium potential within a voltage range of 0.001–2.5 V vs. Na/Na^+^.

## 3. Results and Discussion

### 3.1. Materials Characterizations

**Figure 1:**
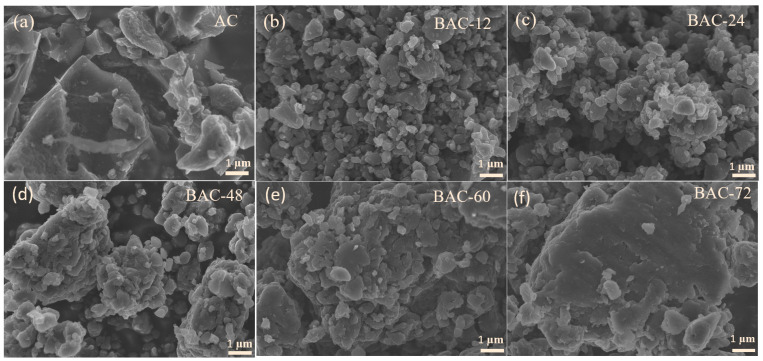
SEM images of (**a**) AC, (**b**) BAC-12, (**c**) BAC-24, (**d**) BAC-48, (**e**) BAC-60, and (**f**) BAC-72.

The precursor of commercial activated carbon YP-50F is coconut shell, sourced from Kuraray Co., Ltd. In Tokyo, Japan. It is widely utilized in the fields of supercapacitors and secondary batteries. Scanning electron microscopy (SEM) was employed to examine the morphology of the activated carbon materials. Figure 1a shows that the non-ball-milled activated carbon (AC) displays a blocky morphology with irregular shapes and relatively loose particles. The ball milling process caused fragmentation of the activated carbon, with particles becoming more spherical and their edges rounding as milling time increased (Figure 1b). This change may indicate a decrease in the surface area. With prolonged ball milling, the particles became more compact, eventually aggregating (Figure 1f), which may prolong the ionic-transport distance and reduce the rate performance. HRTEM images (Figure S2) clearly illustrate the evolution of graphitic structure in BACs. The activated carbon (AC) displayed a highly disordered structure with minimal graphitic regions in the bulk phase. In the BAC-24 samples, small graphitic domains formed due to mechanical ball-milling. Subsequently, large-area graphitic regions developed through the growth and merging of these small domains, accompanied by the elimination of nanopores. The above results indicate that due to the strong impact and shear forces, ball milling, as a straightforward and direct approach to defect engineering, can easily break C=C/C-C bonds, leading to carbon atom rearrangement that transforms disordered structures into graphitic structures. In addition, underscoring the importance of selecting the appropriate milling duration for processing activated carbon. It is noted that high temperatures can facilitate the repair of defects and the elimination of oxygen functional groups. The BAC-60-1400 sample (Figure S2d), post-heat treatment, showed a further increase in the area of ordered graphite domains. The results of TEM indicate a significant enhancement in the degree of graphitization of the material after ball milling and secondary heat treatment.

**Figure 2:**
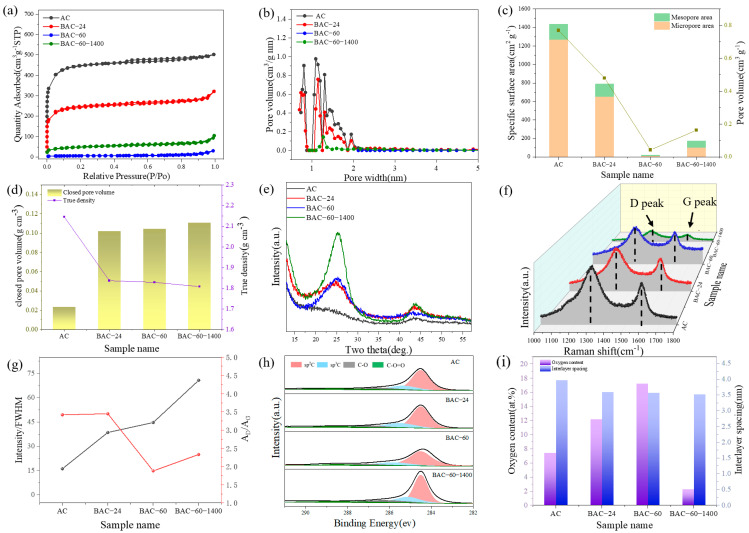
Structural characterization of the ball-milled activated carbon. (**a**) N_2_ adsorption-desorption isotherm; (**b**) Pore size distribution; (**c**) Specific surface area and pore volume; (**d**) Closed pore volume and true density; (**e**) XRD pattern; (**f**) Raman spectrum; (**g**) Variation of intensity/FWHM of the XRD (002) Peak and AD/AG from the Raman spectra of the samples; (**h**) XPS C1s spectrum; (**i**) Oxygen content and layer spacing.

Nitrogen gas adsorption isotherms were utilized to examine the changes in the specific surface area and pore structure of the activated carbon. Figure 2a reveals that the activated carbon (AC) displayed a Type I N_2_ adsorption-desorption isotherm, characterized by rapid adsorption at low relative pressures (<0.1) and a plateau in medium- to high-pressure regions. This pattern indicates a primarily microporous structure with a minor presence of narrow mesopores (Figure 2b). Following mechanical ball milling treatment, the N_2_ adsorption capacity gradually diminished with increased milling time, approaching zero after 60 h. This trend suggests the elimination of open pores. Consequently, the specific surface area and pore volume of the AC declined from 1437 m^2^ g^−1^ and 0.77 cm^3^ g^−1^ to 19.61 m^2^ g^−1^ and 0.042 cm^3^ g^−1^ (Figure 2c). In order to further investigate the changes in pore structure, closed-pore testing was conducted. The closed-pore volume increased from 0.024 to 0.105 cm^3^ g^−1^ (Figure 2d) with ball-milling time. The BAC-60-1400 sample, post-heat treatment, exhibited a slight rise in specific surface area and pore volume compared to BAC-60, as the removal of gases from the bulk phase during heat treatment generated some micropores. However, the closed-pore content remained nearly unchanged. These alterations in pore structure suggest that ball milling for optimal durations is an effective method to reduce the specific surface area of activated carbon while enhancing the closed-pore volume.

Figure 2e presents the XRD spectrum of the AC sample. The (002) and (100) diffraction peaks at approximately 22° and 44° were of low intensity and broad, indicating a highly disordered structure. As ball milling time extended to 24 h, these peaks progressively sharpened and intensified. The (002) peak shifted to a higher angle, around 26°, signifying an increase in *d*_002_ interlayer spacing after ball milling (Figure 2i). The microcrystalline size, specifically lateral size (*L*a) and stack height (*L*c), was determined using the Debye–Scherrer equation [42]. The *L*c _(002)_ values for AC, BAC-24, and BAC-60 were 2.71, 2.46, and 2.43 nm, respectively, while *L*a _(100)_ measured 1.416, 1.423, and 1.433 nm for these samples. To elucidate the change in crystallinity, the ratio of the (002) peak intensity to its full width at half maximum (FWHM) was used (Figure 2g). This ratio increased monotonically from 16.1 for AC to 44.7 for BAC-60, indicating a significant enhancement in the graphitization degree of the activated carbon. The variation in graphitization degree aligns with the TEM.

Similarly, the Raman spectrum (Figure 2f) demonstrated a reduction in the intensity ratio of the D-band to the G-band *(I*_D_/*I*_G_) with increased ball milling time of the activated carbon. It decreased from 1.40 for AC to 1.15 for BAC-60, suggesting a more ordered carbon structure. The area ratio of the D-band to the G-band (*A*_D_/*A*_G_), as depicted in Figure 2g, initially increased from 3.42 for AC to 3.45 for BAC-24, then sharply decreased to 1.88 for BAC-60. The initial rise in defect density was attributed to the breakdown of AC particles, revealing pores and edges. The subsequent ratio decline indicated defect repair through a “disruptive” ball milling process. Overall, Raman testing results signified an increase in material order, aligning with XRD analysis findings.

XPS (X-ray Photoelectron Spectroscopy) is employed to characterize the changes in the surface chemical composition of activated carbon. XPS results showed that ball milling in air introduced numerous oxygen-containing functional groups into BAC, with oxygen content (Figure 2i) rising from 7.6% for AC to 17.3% for BAC-60. These changes will have a significant impact on the initial Coulombic efficiency of the battery [45]. Post-secondary heat treatment, the oxygen content decreased to just 2.29%. This decrease in oxygen content is advantageous for achieving batteries with high reversible capacity. C1s XPS served as another method for analyzing order degree, indicated by changes in the proportion of C(sp^3^) to C(sp^2^) with varying ball milling durations. Figure 2h shows the XPS spectra of activated carbon samples at different ball milling times. The XPS C1s spectrum could be deconvoluted into four peaks at 284.8, 285.2, 286.5, and 289.1 eV, corresponding to sp^2^, sp^3^, C–O, and C-O=C components, respectively [44]. Fitting calculations revealed a gradual increase in sp^2^C content from 38.5 at% for BAC-12 to 55.7 at% for BAC-60, while corresponding sp^3^ C content decreased from 33.8 at% to 13.1 at%. This change indicated the formation of ordered carbon structures, corroborating the earlier Raman results.

### 3.2. Electrochemical Performance

This study demonstrates that a closed-pore structure is beneficial for creating carbon anode materials capable of plateau-stage energy storage, potentially lowering their operating voltage. The research focuses on the physicochemical property alterations of activated carbon during ball milling, aiming to produce carbon anode materials with high specific capacity and low working voltage.

**Figure 3:**
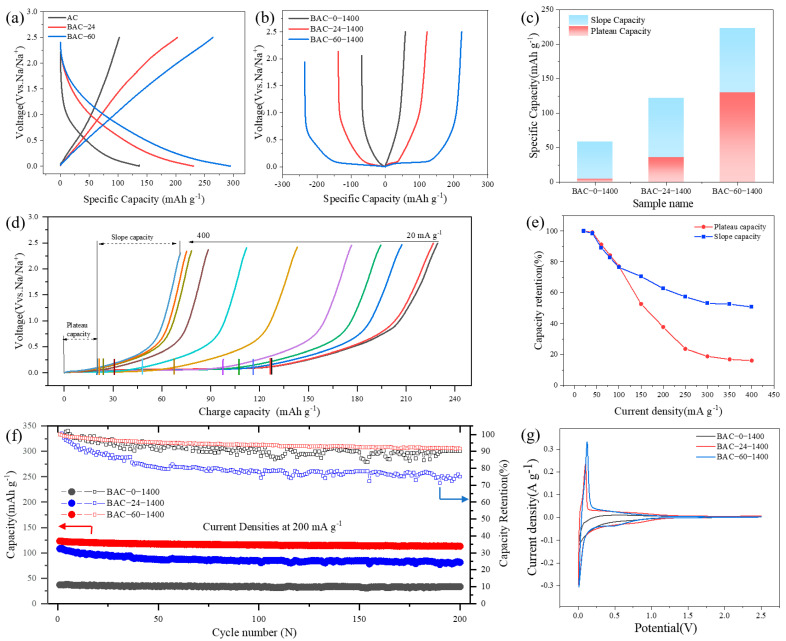
(**a**,**b**) Constant current charge and discharge curve of an activated carbon electrode at a 0.1C current rate; and (**c**) Specific capacity from the plateau (<0.1 V) and slope (>0.1 V) contributions (based on the second discharge/charge curves). (**d**) Charge curves at varied current densities from 20 to 400 mA g^−1^, and (**e**) statistical results of plateau and slope capacity in inset (**d**). (**f**) long-term cycling performance at 200 mA g^−1^. (**g**) CV curves of BAC-0-1400, BAC-24-1400, and BAC-60-1400 when the scan rate is 0.1 mV s^−1^.

The charge-discharge curves of the ball-milled activated carbon electrode without secondary heat treatment are shown in Figure 3a. The reversible capacity of AC is 102 mAh g^−1^, while the reversible capacity of BAC-60 is 264 mAh g^−1^. It is evident that the reversible capacity of the battery increases with the extension of the ball-milling time for the activated carbon sample. However, no plateau segment is observed. Examining the XPS test results (Figure 2i) and comparing them with AC, the oxygen content of ball-milled activated carbon is significantly higher. This is attributed to ball milling in the presence of air, which introduces a large number of oxygen-containing functional groups into the activated carbon matrix. This measure not only provides more active sites for ball-milled activated carbon but also enhances the material’s specific capacity, thereby promoting an increase in the reversible capacity of ball-milled activated carbon.

Figure 3b shows the constant current charge-discharge curves of activated carbon electrodes after 1400 °C heat treatment for different ball-milling times. It can be seen that the non-ball-milled activated carbon electrode still does not exhibit a low-voltage plateau, and when ball-milled for 24 h, the voltage plateau begins to appear. At a current rate of 0.1C, the BAC-60-1400 electrode maintains a high reversible capacity (225 mAh g^−1^) while exhibiting a low-voltage plateau, with the capacity proportion of the plateau exceeding that of the slope segment. After secondary heat treatment, the oxygen content of the sample is greatly reduced, and the corresponding slope capacity is significantly decreased. However, due to the effective elimination of defects and an increase in graphitization degree, as well as an increase in closed-pore volume, the corresponding electrode’s platform capacity is greatly increased. A comparison between Figure 3a,b reveals that mechanical ball milling is an effective method to increase the battery capacity and platform capacity.

Figure 3c further compares the slope capacity and plateau capacity of the activated carbon electrode (Figure 3b). With increasing ball-milling time, the proportion of the slope segment in the constant current charge-discharge curve gradually decreases, while the proportion of the plateau segment increases. The platform capacity and the corresponding proportion of BAC-60-1400 reach 126 mAh g^−1^ and 56%, respectively. This difference highlights the impact of ball milling on the sodium-ion mechanism.

This study also identifies key factors influencing the rate performance of the activated carbon electrode by statistically analyzing the capacities of plateau and sloping segments in the charge-discharge curves of the BAC-60-1400 electrode at varying current densities (Figure 3d). As Figure 3e demonstrates, beyond 100 mA g^−1^, the plateau capacity sharply declines linearly, whereas the sloping capacity remains relatively stable. These findings suggest that at higher current densities, sodium ions are unable to quickly infiltrate the closed-pore structure. Hence, the superior high-rate performance of the ball-milled activated carbon electrode is ascribed to the preserved contribution of surface-controlled capacity with increasing current density. Concurrently, the ball-milled activated carbon electrode exhibits remarkable cycling performance (Figure 3f). Maintaining a capacity retention rate of 92% after 200 cycles at 200 mA g^−1^, the electrode’s durability over extended cycles is highlighted.

Cyclic voltammetry (CV) is a crucial technique for assessing electrochemical properties. Figure 3g illustrates the CV curves of electrodes with different ball milling durations in a voltage range of 0 to 2.5 V (vs. Na^+^/Na) at a scan rate of 0.1 mV s^−1^. The CV curves reveal prominent peaks around 0.1 V, associated with the insertion/extraction process of sodium in the carbon material’s intermediate layer. A weak “camel hump” between 0.2 and 1.0 V is attributed to pseudo-adsorption or surface defects caused by Na, correlating with the plateau and sloping regions in the charge-discharge curves. Additionally, a broad reduction peak around 0.5 V is noted in the CV curves (Figure S4). The BAC-60-1400 electrode’s broad reduction peak area is smaller than that of BAC-24-1400, while the area around 0.1 V is larger, suggesting that BAC-60-1400 requires less energy for SEI film formation, likely due to more ordered carbon structures formed during longer ball milling. Electrochemical impedance spectroscopy (EIS), presented in Figure S5, comprises two regions: a high-frequency semicircle representing resistance to charge transfer between the electrolyte and electrode, and a low-frequency linear region denoting Warburg resistance associated with Na^+^ ion diffusion. The noticeable reduction in the high-frequency semicircle of the activated carbon post-ball milling indicates reduced resistance, thus facilitating faster charge transfer.

### 3.3. Electrochemical Analysis

**Figure 4:**
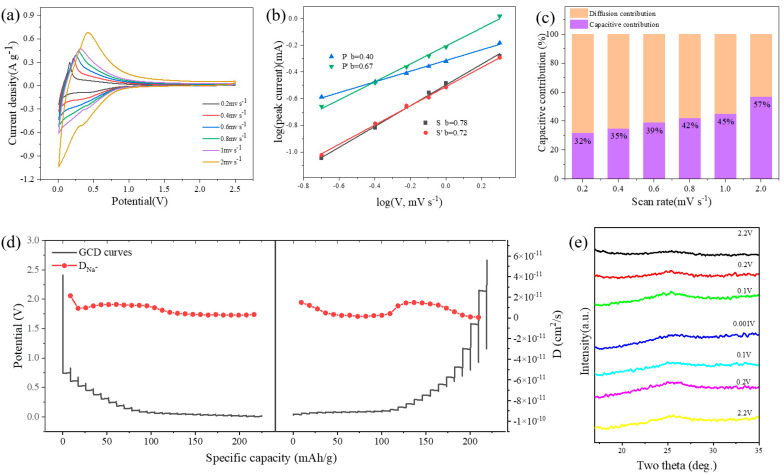
(**a**) CV curve of BAC-60-1400 from 0.2 to 2 mV s^−1^. (**b**) The relationship between the peak current and the scanning rate at which the b value is determined. (**c**) The percentage contribution of capacitive and diffusive dominant capacity at different scan rates. (**d**) GITT test results of the BAC-60-1400 electrodes. (**e**) Ex situ XRD profiles of BAC-60-1400 electrodes at various stages of sodiation and desodiation.

To investigate the sodium storage mechanism in ball-milled activated carbon electrodes, CV tests were conducted at different scan rates in a half-cell. Figure 4a reveals the CV measurements of BAC-60-1400 at various scan rates to assess rate behavior. At elevated scan rates, the CV curves deviate from standard positions, indicating increased polarization and ohmic resistance. The equation i = av^b^ is employed to explore the correlation between the measured current (i) and scan rate (v), facilitating the determination of diffusion and capacitance contributions to the electrochemical behavior. The value of b, indicative of the dominant process (surface-controlled for values near 1 and diffusion-controlled for values around 0.5), is derived from the slope of the log(v)–log(i) plot. As evidenced in Figure 4b, which illustrates the log(i) vs. log(v) plots and fitting results for peaks P/P′ and S/S′, the calculated b values for P/P′ and S/S′ peaks are 0.40/0.67 and 0.78/0.72, respectively. This suggests that the plateau region below 0.2 V in the GCD curve, corresponding to the P/P′ peak range in CV, is associated with the slow filling of sodium in closed pores. Conversely, the high-potential sloping region of the GCD curve relates to sodium extraction/insertion from the graphene interlayer in graphite nanodomains. At a scan rate of 0.2 mV s^−1^, the CV curve shows a capacitive capacity constituting 32% of the total capacity, whereas at 2 mV s^−1^, capacitive processes predominantly govern the electrochemical behavior.

The sodium diffusion coefficients during sodiation are obtained via the Galvanostatic Intermittent Titration Technique (GITT), offering further insight into the Na^+^ storage mechanism in the BAC-60-1400 anode. This method enables the analysis of electrode kinetics throughout the charge-discharge cycle. Figure 4c presents the GITT curves for BAC-0-1400 and BAC-60-1400 electrodes at a 0.1C current rate, with Na^+^ diffusion coefficients (D_Na+_) calculated according to Fick’s second law. During Na^+^ insertion, D_Na+_ notably decreases across all samples. This indicates slower electrochemical Na^+^ filling in the plateau region (<0.1 V, bulk diffusion) compared to the faster diffusion kinetics in the sloping region. The differential dynamics stem from faster Na^+^ storage through insertion in the high-potential sloping region and slower kinetics in the low-potential plateau region, primarily involving Na^+^ deposition in closed pores.

The widely acknowledged sodium storage mechanisms for hard carbon are mainly divided into varied types, including “adsorption-intercalation mechanism”, “adsorption-pore filling mechanism”, and “intercalation-pore filling mechanism” [46,47,48], which can be identified by monitoring the changes of microstructure during the charging/discharging processes. Ex-situ XRD testing has been conducted to analyze the sodium storage mechanism of the BAC electrode in this paper (Figure 4e). Discharge from the open-circuit voltage to 0.2 V, 0.1 V, and 0.001 V, followed by charging to 0.10 V, 0.2 V, and 2.2 V, is displayed in Figure 4e. The gradual discharge of the electrode from 2.2 V to 0.1 V causes a leftward shift in the (002) peak and an increase in peak intensity. The corresponding interlayer spacing *d*_002_ increased from 3.49 Å at 2.2 V to 3.51 Å at 0.1 V. During discharge from 0.1 V to 0.001 V, the (002) peak position remains constant, and the interlayer spacing *d*_002_ diminishes to 3.45 Å. After charging to 2.2 V, the (002) peak largely reverts to its original position. The interlayer spacing of the material was less than 0.4 nm, making it challenging for the slope segment capacity to be influenced by the adsorption mechanism [49]. Simultaneously, when the material stores sodium ions through ion adsorption at the surface active sites, *d*_002_ remains unchanged. However, when storing sodium through sodium insertion, *d*_002_ gradually increases [50]. In this experiment, as the discharge process progresses with voltages greater than 0.1 V, the peak position gradually shifts to the left, indicating an increase in interlayer spacing. At voltages less than 0.1 V, the peak position remains unchanged, and there is a decrease in interlayer spacing. Based on these observations, the sodium storage mechanism for ball-milled activated carbon in this experiment is identified as the “insertion-pore filling mechanism”. During discharge, Na insertion predominantly occurs in the sloping portion, followed by reduction to quasi-metallic Na clusters that fill the closed pores in the low-voltage plateau region.

## 4. Conclusions

In this study, ball milling was employed to adjust the microstructure of activated carbon to enhance sodium storage capacity. Post-ball milling, there is a notable increase in closed-pore volume from 0.024 to 0.105 cm^3^ g^−1^ and a significant enhancement in the graphitized structure. Consequently, the sodium storage capacity of the platform segment of activated carbon exhibits considerable growth. Furthermore, analyses using the galvanostatic intermittent titration technique (GITT) and X-ray diffraction (XRD) at varied potentials reveal that the sodium storage process in the modified activated carbon adheres to an embedment-pore filling mechanism. Above results demonstrate that ball-milling is an effective method for altering the microstructure of carbon materials. This provides a novel strategy to prepare the anode of a sodium-ion battery. Further enhancements could be achievable through the control of surface chemical property transformations during ball milling or the selection of appropriate precursor materials and the construction of their pore structures. These findings hold significant potential for the advancement of high-performance hard carbon materials suitable for practical sodium-ion batteries.

## Data Availability

Data are contained within this article.

## Supplementary Materials

The following supporting information can be downloaded at: https://www.mdpi.com/article/10.3390/nano14010065/s1. Figure S1: SEM images of (a) BAC-0-1400, (b) BAC-24-1400, and (c) BAC-60-1400; Figure S2: TEM images of (a) AC, (b) BAC-24, (c) BAC-60, and (d) BAC-60-1400; Table S1: Physical parameters for the obtained samples; Figure S3: (a,b) Charge and discharge curve of the first ring and IEC of the activated carbon electrode at a 0.1C current rate; Figure S4. CV curves of (a) BAC-0-1400, (b) BAC-24-1400, and (c) BAC-60-1400 when the scan rate is 0.1 mV s^−1^; Figure S5: EIS of BAC-0-1400, BAC-24-1400, and BAC-60-1400; Figure S6: GITT test results of the BAC-0-1400 electrodes. Figure S7: Ex situ XRD profiles of BAC-0-1400 electrodes at various stages of sodiation and desodiatio.
