# Supplementary material for: Enhancing Sodium-Ion Energy Storage of Commercial Activated Carbon by Constructing Closed Pores via Ball Milling
Source: Nanomaterials (Basel). 2023 Dec 26;14(1):65. doi: 10.3390/nano14010065 (PMC10780331; doi:10.3390/nano14010065)
Supplement: Supplementary file 1 [file nanomaterials-14-00065-s001.zip › nanomaterials-2781597-supplementary.pdf]

Supporting Information

Enhancing Sodium-Ion Energy Storage of Commercial Activated Carbon by Constructing Closed Pores via Ball Milling

Xiaojie Wang, Qian Fang, Tiejun Zheng, Yanyan Xu, Rui Dai, Zhijun Qiao, Dianbo Ruan * and Yuzuo Wang *

Institute of Advanced Energy Storage Technology and Equipment Faculty, Ningbo University,
Ningbo 315211, China; 17806230316@163.com (X.W.); afangqian@163.com (Q.F.); stress1224@163.com (T.Z.); 15856717889@163.com (Y.X.); 17754110986@163.com (R.D.); qiaozhi8702@163.com (Z.Q.)
* Correspondence: ruandianbo@nbu.edu.cn (D.R.); wangyuzuo@nbu.edu.cn (Y.W.)

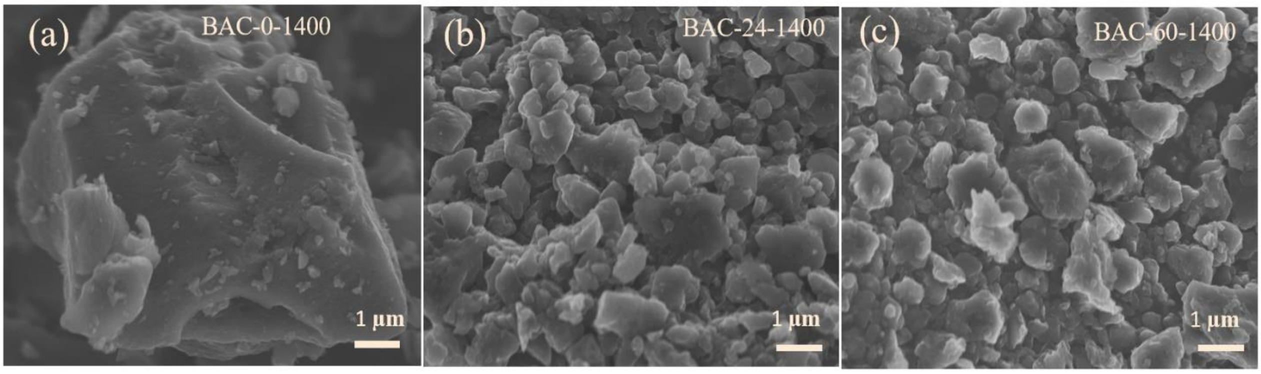

Figure S1. SEM images of (**a**) BAC-0-1400, (**b**) BAC-24-1400, (**c**) BAC-60-1400

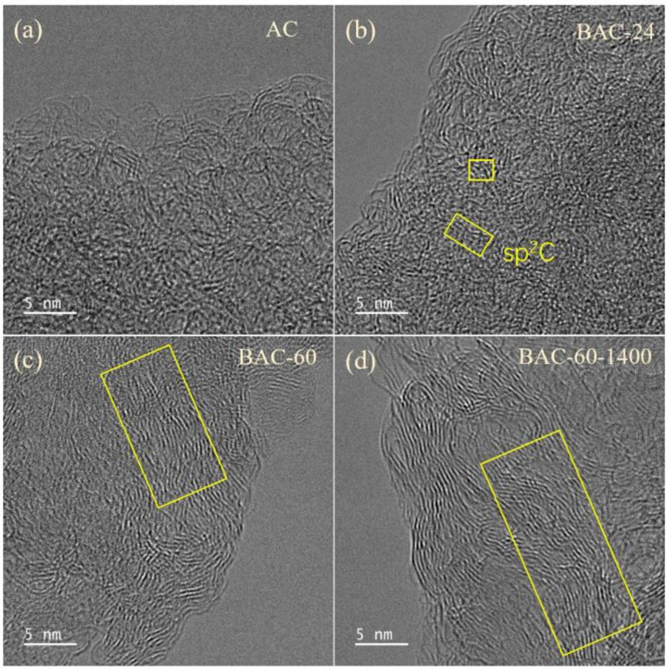

Figure S2. TEM images of (a) AC, (**b**) BAC-24, (**c**) BAC-60 and (**d**) BAC-60-1400.

Samples	AC	BAC-24	BAC-60	BAC-60-1400
true density (g cm^{-3})	2.145	1.836	1.828	1.808
closed pore volume (cm^3 g^{-1})	0.024	0.102	0.105	0.111
d_{002} (nm)	3.96	3.60	3.56	3.49
Lc (nm)	2.71	2.46	2.43	2.39
La (nm)	1.416	1.423	1.433	1.431
SSA (m^2 g^{-1})	1437	788	19.61	173

Table S1. Physical parameters for the obtained samples.

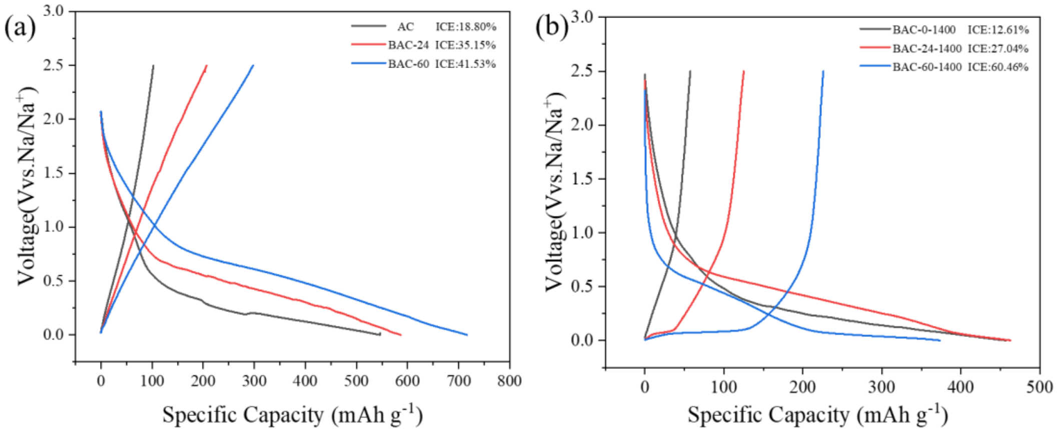

Figure S3. (**a**), (**b**) Charge and discharge curve of first ring and IEC of activated carbon electrode at 0.1C current rate

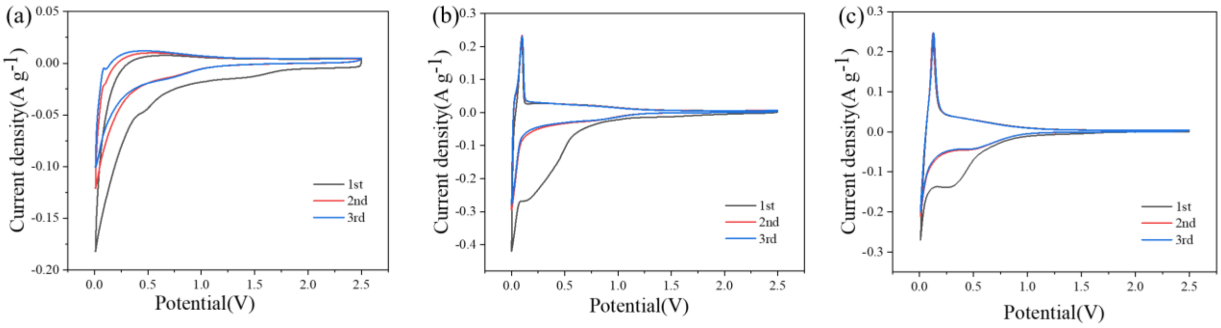

Figure S4. CV curves of (**a**) BAC-0-1400, (**b**) BAC-24-1400 and (**c**) BAC-60-1400 when the scan rate is 0.1 mV s−1

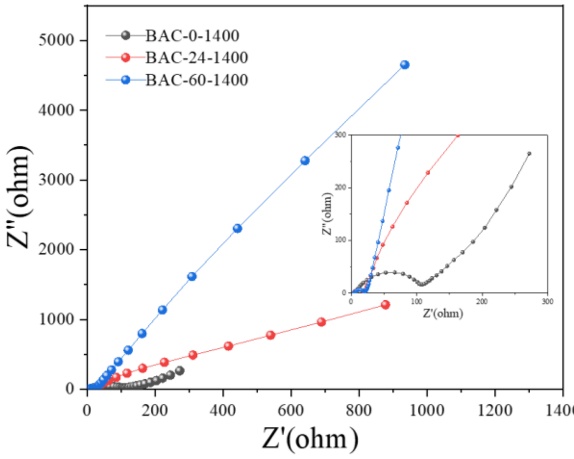

Figure S5.EIS of BAC-0-1400, BAC-24-1400, BAC-60-1400

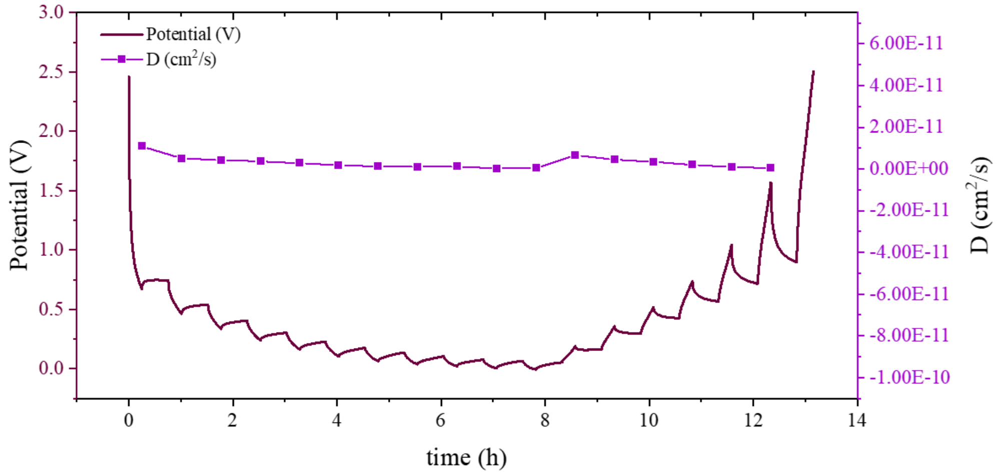

Figure S6 GITT test results of the BAC-0-1400 electrodes.

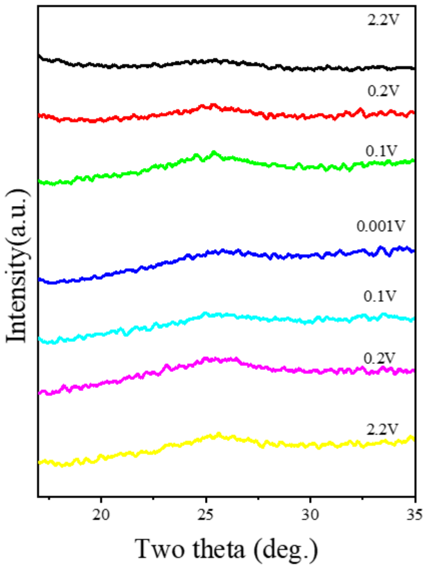

Figure S7 Ex situ XRD profiles of BAC-0-1400 electrodes at various stage of sodiation and desodiatio.
